# Supplementary material for: Intermittent Theta Burst Stimulation Improves Motor and Behavioral Dysfunction through Modulation of NMDA Receptor Subunit Composition in Experimental Model of Parkinson’s Disease
Source: Cells. 2023 Jun 1;12(11):1525. doi: 10.3390/cells12111525 (PMC10252812; doi:10.3390/cells12111525)
Supplement: Supplementary file 1 [file cells-12-01525-s001.zip › Suplementary figure S1 legend .pdf]

Suplementary Figure S1. Representative images of MRI rat's brain scan

Representative images of MRI rat's brain scan depicting caudoputamen (CPu) and substantia nigra *pars compacta* (SNpc) in sagital (A), coronal (B) and transversal anatomical plane (C)
