# Supplementary figures and images for: Intermittent Theta Burst Stimulation Improves Motor and Behavioral Dysfunction through Modulation of NMDA Receptor Subunit Composition in Experimental Model of Parkinson’s Disease
Source: Cells. 2023 Jun 1;12(11):1525. doi: 10.3390/cells12111525 (PMC10252812; doi:10.3390/cells12111525)

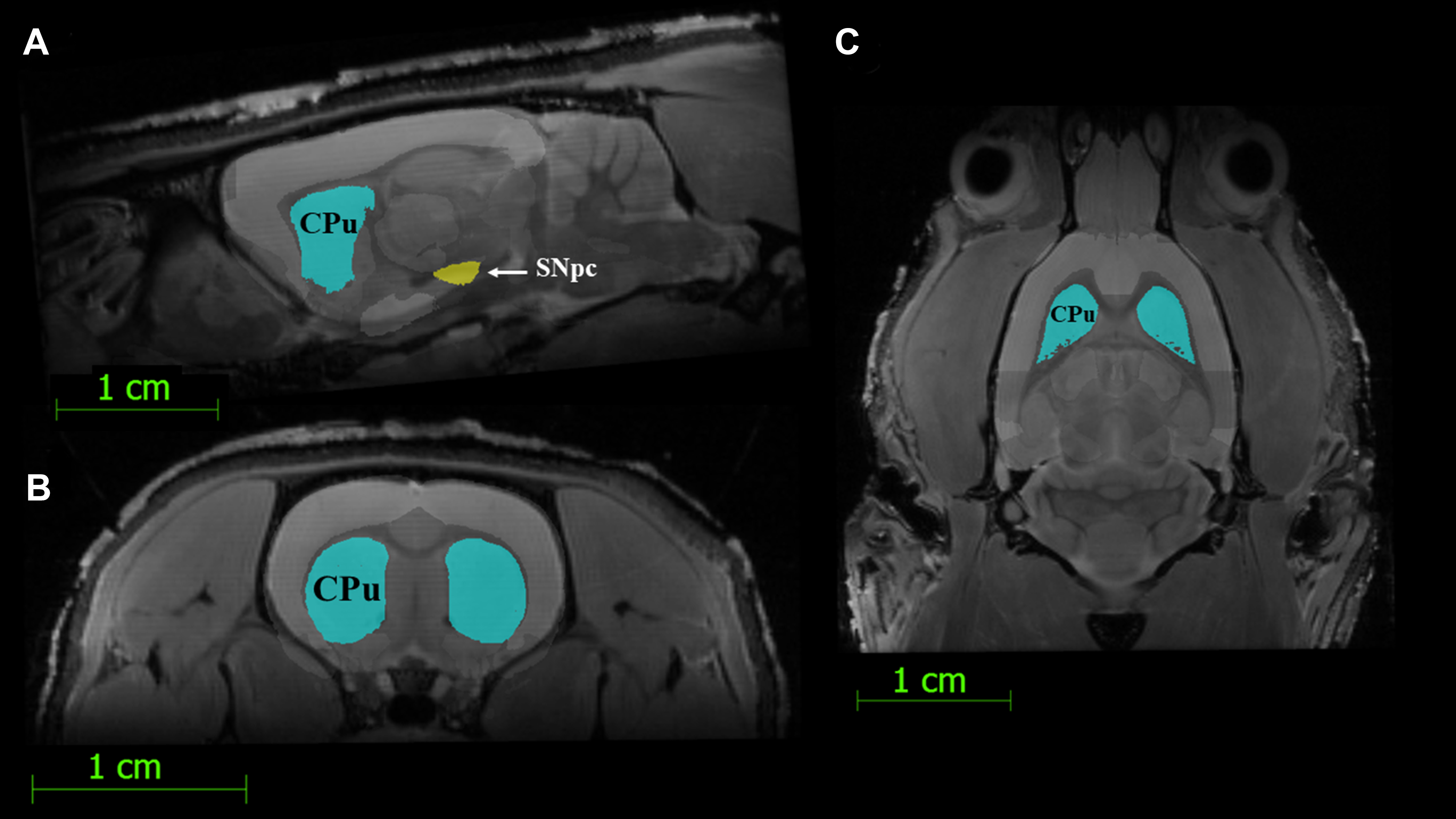

Supplement: Supplementary file 1 [file cells-12-01525-s001.zip › Suplementary Figure S1.tif]
